# Intrauterine inflammation and postnatal intravenous dopamine alter the neurovascular unit in preterm newborn lambs

**DOI:** 10.1186/s12974-024-03137-0

**Published:** 2024-05-28

**Authors:** Nhi T. Tran, Nadia Hale, Anawar Aung Win Maung, Manon Wiersma, David W. Walker, Graeme Polglase, Margie Castillo-Melendez, Flora Y. Wong

**Affiliations:** 1 The Ritchie Centre, The Hudson Institute of Medical Research, Melbourne, Australia; 2 Department of Obstetrics and Gynaecology, Monash University, Melbourne, Australia; 3 Department of Paediatrics, Monash University, Melbourne, Australia; 4 Monash Newborn, Monash Medical Centre, Melbourne, Australia; 5 Monash Children’s Hospital, Level 5, 246 Clayton Rd, Clayton, VIC 3168 Australia

**Keywords:** Intrauterine inflammation, Chorioamnionitis, Dopamine, Preterm brain, Neurovascular unit, Neurovascular coupling

## Abstract

**Background:**

Intrauterine inflammation is considered a major cause of brain injury in preterm infants, leading to long-term neurodevelopmental deficits. A potential contributor to this brain injury is dysregulation of neurovascular coupling. We have shown that intrauterine inflammation induced by intra-amniotic lipopolysaccharide (LPS) in preterm lambs, and postnatal dopamine administration, disrupts neurovascular coupling and the functional cerebral haemodynamic responses, potentially leading to impaired brain development. In this study, we aimed to characterise the structural changes of the neurovascular unit following intrauterine LPS exposure and postnatal dopamine administration in the brain of preterm lambs using cellular and molecular analyses.

**Methods:**

At 119–120 days of gestation (term = 147 days), LPS was administered into the amniotic sac in pregnant ewes. At 126-7 days of gestation, the LPS-exposed lambs were delivered, ventilated and given either a continuous intravenous infusion of dopamine at 10 µg/kg/min or isovolumetric vehicle solution for 90 min (LPS, *n* = 6; LPS_DA_, *n* = 6). Control preterm lambs not exposed to LPS were also administered vehicle or dopamine (CTL, *n* = 9; CTL_DA_, *n* = 7). Post-mortem brain tissue was collected 3–4 h after birth for immunohistochemistry and RT-qPCR analysis of components of the neurovascular unit.

**Results:**

LPS exposure increased vascular leakage in the presence of increased vascular density and remodelling with increased astrocyte “end feet” vessel coverage, together with downregulated mRNA levels of the tight junction proteins Claudin-1 and Occludin. Dopamine administration decreased vessel density and size, decreased endothelial glucose transporter, reduced neuronal dendritic coverage, increased cell proliferation within vessel walls, and increased pericyte vascular coverage particularly within the cortical and deep grey matter. Dopamine also downregulated *VEGFA* and Occludin tight junction mRNA, and upregulated dopamine receptor *DRD1* and oxidative protein (*NOX1, SOD3*) mRNA levels. Dopamine administration following LPS exposure did not exacerbate any effects induced by LPS.

**Conclusion:**

LPS exposure and dopamine administration independently alters the neurovascular unit in the preterm brain. Alterations to the neurovascular unit may predispose the developing brain to further injury.

**Supplementary Information:**

The online version contains supplementary material available at 10.1186/s12974-024-03137-0.

## Background

Intrauterine infection or inflammation which manifests as chorioamnionitis is a major contributor to preterm cerebral injury leading to adverse neurodevelopmental outcomes along with long-term disabilities [1–4]. Dysregulation of cerebral blood flow (CBF) occurs alongside neuropathology in chorioamnionitis [5]. Preterm lambs and infants with chorioamnionitis have increased basal CBF and cerebral oxygen delivery [6, 7], likely due to the neuroinflammation. But they can have an even greater and disproportionate increase in cerebral oxygen consumption and oxygen extraction [7], indicating a baseline mismatch between the cerebral oxygen delivery and oxygen consumption. We have reported that preterm lambs exposed to intrauterine inflammation, induced by intra-amniotic injection of lipopolysaccharide (LPS), are more likely to show a negative cerebral functional haemodynamic response (i.e., localised decrease in oxyhaeomoglobin) suggesting that neuronal activity, which increases the demand for oxygen, is likely to create localized oxygen deficits [8].

Increased local neuronal activity normally leads to increases of local cerebral perfusion and oxygenation to meet the increased local metabolic demand – a physiological response known as neurovascular coupling [9]. Tight coupling of cerebral function, metabolism, and blood flow has been demonstrated in numerous physiological, biochemical, and clinical studies [10]. A complex network of neurons and astrocytes are in close physical proximity with, and functionally coupled to the basal lamina, pericytes, smooth muscle cells, and the endothelial cells of cerebral arterioles. The interconnection and interactions of these cells constitute the “neurovascular unit” (NVU) [11]. This unique vascular coupling system regulates blood flow, supports brain growth and homeostasis, and coupled with the development of the blood-brain barrier (BBB), the NVU also enables clearance of toxic byproducts of brain metabolism and immune surveillance.

Relatively little research has looked specifically at the NVU in the developing brain and perinatal period, despite studies suggesting differences in NVU structure and/or function between the developing and adult brain. Importantly, perinatal insults associated with adverse neurodevelopmental deficits, such as hypoxic-ischemia or infection such as chorioamnionitis, are typically associated with changes in both CBF and the presence of neuropathology, suggesting that neurovascular development may be critically affected. Notably, inflammation has long been known to promote disruption of the BBB and neurovasculature in both the mature and immature brain [11–13]. Given the adverse long-term consequence of chorioamnionitis, insights into the neurovascular effects of intrauterine inflammation in the preterm brain, particularly on the NVU, will be important for understanding the events that lead to brain injury, dysfunction, and repair, as well as identification of therapeutic targets. In addition, we have previously reported that infusion of the inotrope dopamine, often required to treat hypotensive preterm infants following infection/inflammation, increases the baseline cerebral oxygenation but also further increases the incidence of negative functional haemodynamic responses in preterm lambs exposed to intrauterine inflammation [8, 14]. To fully understand the implications of these effects on neurovascular coupling, histopathological analysis of the NVU would provide mechanistic insights at the cellular and molecular levels.

In the present study, we investigated the effects on the cellular and molecular components of the NVU in preterm newborn lambs previously exposed to intra-amniotic LPS. We also determined the effects of postnatal dopamine infusion on the NVU with and without exposure to this intrauterine inflammation. We hypothesized that exposure to intrauterine inflammation alters the cerebral vasculature in the brain of preterm lambs so that neurovascular coupling is obtunded, and that dopamine treatment further exacerbates these changes.

## Methods

### Experimental procedures

This study utilized post-mortem preterm lamb brain tissue collected after in vivo physiological studies as previously published [8]. The use of animals was approved by Monash Medical Centre Animal Ethics Committee (MMCA-2016/17 and 2016/23) and was conducted in accordance with the Australian Code of Practice for the care and use of Animals for Scientific Purposes established by the National Health and Medical Research Council of Australia.

Time-mated pregnant ewes (Merino-Border Leicester cross) used in this study were supplied by the Monash Animal Research Platform. At 119–120 days of gestation (dGA; Term is ~ 147 dGA), pregnant ewes received an ultrasound-guided intra-amniotic injection of lipopolysaccharide (LPS; E Coli 055: B5, 10 mg/mL) or saline (control) to induce intra-uterine inflammation and elicit the multi-system complications of chorioamnionitis observed in human infants as reported previously [15]. At 126–7 dGA, ewes were anaesthetised by intravenous injection of sodium thiopental (20 mg/kg; Jurox, NSW, Australia), followed by tracheal intubation, positive pressure ventilation and inhaled anaesthesia of isofluorane 1.5–3.5% in oxygen air, and the lambs (*n* = 28) were delivered by caesarean section. Each lamb was weighed, intubated and ventilated in volume-guaranteed mode at ~ 5–7 ml/kg (Babylog8000Plus; Draeger; Germany) while anaesthesia was maintained. Prophylactic surfactant was administered via the endotracheal tube (100 mg/kg Curosurf; ChiesiPharma; Italy). After delivery of the lamb, the ewe was killed by intravenous injection of pentobarbitone (~ 100 mg/kg; pentobarbitone sodium 325 mg/ml, Virbac, Milperra, NSW, Australia).

A pulse oximeter probe was placed on the lamb’s right forelimb or tail to measure SpO_2_ (Radical 4, Masimo Frenchs Forest NSW, Australia). Polyvinyl catheters containing heparinised saline were placed into the umbilical vein and artery, or the jugular vein and carotid artery, to allow continuous measurement of mean arterial blood pressure (MABP) and heart rate (HR) (DTX Plus Transducer; Becton Dickinson), for withdrawing blood for regular blood-gas analysis (ABL30; Radiometer, Copenhagen, Denmark) and for administration of 10% glucose (at 3 ml/kg/h) as fluid maintenance. Ventilatory settings and fractional inspired oxygen were adjusted to maintain SpO_2_ at > 90% and PaCO_2_ at 45–55 mmHg.

Thirteen lambs across the Control and LPS groups were randomly allocated to receive a continuous intravenous infusion of dopamine at 10 µg/kg/min (mid-clinical dose used in hypotensive preterm infants [16]) in heparinised saline for 90 min. The groups were: control, (CTL; *n* = 9), LPS, (LPS; *n* = 6), control and dopamine (CTL_DA_; *n* = 7) or LPS and dopamine (LPS_DA_; *n* = 6). All lambs were ventilated for 3–4 h and then were killed by intravenous injection of pentobarbitone at 100 mg/kg.

### Post-mortem tissue preparation

Brains were transcardially perfused in situ with isotonic saline, then removed, weighed and divided longitudinally at the mid-sagittal plane into the 2 cerebral hemispheres. The right hemisphere was dissected for sampling of cortical grey matter (cortex), white matter (WM; combined sampling from the subcortical and periventricular white matter) and striatum (combined sampling from the putamen and caudate nucleus); samples were snap frozen in liquid nitrogen and stored at -80 °C for mRNA analyses. The left hemisphere was sectioned coronally into 10-mm-blocks for immersion fixation in chilled 4% paraformaldehyde (0.1 M; pH 7.4) for 3 days before paraffin embedding. Coronal brain sections (8 μm) were cut at the level posterior to the ansate sulcus which corresponds to the somatosensory cortex (the sheep brain atlas section number 600, Michigan sheep atlas; [17]). Two coronal brain sections that were 80 μm apart were used for each immunohistochemical marker and results were averaged between the sections for each lamb.

### Single-label immunohistochemistry

Immunohistochemical labelling was conducted using primary antibodies: rabbit anti-NeuN (1:200; Abcam, UK; CAT#: 177487) for mature neurons; rabbit anti-microtubule-associated protein 2 (MAP2; 1:250; Sigma-Aldrich, USA; CAT#:M3696) for neuronal dendrites; rabbit anti-sheep serum (1:700; Sigma-Aldrich, USA; CAT#:S4265) for serum protein extravasation to detect vascular leakage; rabbit anti-matrix metallopeptidase 9 (MMP9; 1:200; Thermo Fisher Scientific, USA; CAT#:RB-1539-P) as a marker for inflammation and basement membrane integrity; rabbit anti-glucose transporter 1 (GLUT1; 1:250; Abcam, UK; CAT#:ab14683) to identify mature endothelial cells and; rabbit anti-Ki67 (1:100; Thermo Fisher Scientific, USA; CAT#:MA5-14520) for proliferating cells. Briefly, tissue sections were de-paraffinized, then pre-treated for antigen retrieval with citrate buffer (10 mM Tri-sodium citrate in dH_2_O, pH 6.0; Sigma Aldrich), PBS washes, endogenous peroxidase blocking, and blocked with serum free protein block (Code X090, Dako, USA). Primary antibodies were incubated overnight at 4°C in Dako antibody diluent (Code S3022, Dako, USA). Sections were incubated in secondary goat biotinylated anti-rabbit IgG for 2 h (1:200; Vector Laboratories, UK; CAT#: BA-100) then incubated with avidin-biotin complex (ABC Elite kit; 1:1:200 in PBS; Vectastain®, Vector Laboratories, UK) and visualized with 3,3’-diamniobenzidine solution (DAB; MP Biomedicals, USA). Ki67 and sheep serum antibody labelling were counter-stained with haemotoxylin. Sections were digitally scanned (Image Scope, Aperio Technologies Inc., Germany) and analysed at 40x magnification.

### Double-label immunohistochemistry

To identify astrocytes associated with blood vessels, sections were incubated in mouse monoclonal anti-GFAP (1:500; Sigma, USA; CAT#:G3893-.2ML) and rabbit anti-laminin (1:200; Novus Biologicals, USA; CAT#:NB300-144). To identify pericytes, sections were incubated in mouse monoclonal α-smooth muscle actin (α-SMA, 1:50; Sigma, USA; CAT#:A5228) and rabbit polyclonal anti-desmin (1:50; Sigma, USA; CAT#:D8281). Tissue sections were de-paraffinized, pre-treated for antigen retrieval, then washed with sodium borohydride (10 mg/ml) in 0.1 M PBS to reduce autofluorescence. Sections were then blocked with serum free protein block and primary antibodies incubated overnight at 4 °C in Dako antibody diluent. Immunoreactivity was visualized using the appropriate secondary antibodies for each primary antibody with Alexa Fluor®488 goat anti-rabbit (1:500, Invitrogen, USA) and Alexa Fluor®594 goat anti-mouse (1:500, Invitrogen, USA). Sections were coverslipped using an aqueous mounting media (code S3023; Dako, California, USA), viewed and images obtained with a Nikon C1 Digital Eclipse Modular Confocal Microscope System (Nikon Instruments Inc. Japan) at 20x magnification for analyses.

### Immunohistochemistry quantification

All analyses were undertaken by investigators blinded to the treatment groups. Regions of interest included the cortical grey matter (cortex), subcortical white matter (SCWM), periventricular white matter (PVWM) and caudate. Within the cortex, 2 non-overlapping fields of view (FOV; 0.04 mm^2^) were placed within each of the 6 gyri (see Supplementary Fig. 1) (except for MAP2, see below) and statistical analysis (one-way ANOVA) was undertaken within each group to assess whether there was an effect of gyri. No significant effect of gyri was found for any of the parameters and thus data for all gyri were averaged for each animal. For SCWM, 4 FOVs were placed within each of the first 4 gyri medial to the base of the gyri. A significant effect of gyri was also not found for the SCWM and thus data for gyri were averaged for each animal. For the PVWM and caudate, 4 FOVs in each region were used for analysis.

NeuN and vascular Ki67 were quantified using immunopositive cell densities. For NeuN, manual cell counts were conducted in FOVs placed within cortical layers IV and V. Pyknotic cells were excluded from neuronal cell counts. For Ki67, the vascular immunopositive cells was expressed as the number of positive cells divided by the perimeter of vessels identified within FOVs.

For MAP2, GFAP and laminin, area coverage within FOVs was quantified using ImageJ software (FIJI, National Institutes of Health, USA), where a set threshold was optimised for detection of positive labelling in FOVs for each antibody respectively. For MAP2 analysis, area coverage was quantified within cortical layers IV, V and VI, in order to capture both basal and apical dendrites, using 4 FOVs in each of the 6 gyri. No differences were found across gyri and cortical layers within each group; thus the area coverage for each of the 24 FOVs were averaged. For GFAP and laminin analysis, laminin-positive area coverage was analysed by viewing only the green fluorescence channel as a measure of vascular density; and GFAP-positive area coverage was analysed by viewing only the red fluorescence channel to assess astrogliosis. For quantitative assessments of area coverage within vessels (MMP9, GLUT1) in each FOV, blood vessel walls were traced in which area coverage analyses were applied as described above and expressed as percentage of the vessel area.

Double label analysis of GFAP-positive immunolabelling and laminin-positive blood vessel walls were measured to quantify astrocyte attachment to blood vessels and; desmin-positive and αSMA-positive blood vessel walls were measured to assess vascular pericyte density. Both analysis were expressed as a percentage of colocalization within blood vessels at x20 magnification, as previously described [18].

Analysis of sheep serum was conducted as previously described [19]. Briefly, blood vessels with sheep serum extravasation were manually identified across the total cross-sectional area of each region of interest and examined for positive immunoreactivity in the surrounding parenchyma. The number and proportion of vessels exhibiting serum extravasation for each brain region were averaged across the lambs in each experimental group.

To assess vascular morphology, vessel perimeter was analysed by measuring the external circumference of only cross-sectional vessels as identified using laminin-positive immunolabelling. A minimum of ~ 10 vessels per FOV was measured, and the average perimeter per vessel per brain region per animal was calculated.

### RT-qPCR

Gene expression for 14 genes relating to vasculature integrity, dopamine and oxidative stress were assessed (Table 1). RNA extraction, cDNA preparation and analysis was conducted as described previously [19]. Briefly, RNA was extracted from frozen brain tissue (60–80 mg) using an RNA extraction kit (RNeasy Midi Kit, Qiagen, Germany). RNA yield was determined by spectrophotometry (Nanodrop, Analytical Technologies, Biolab) then transcribed to cDNA, and pre-amplified to 50 ng/µl (SuperScript® III First-Strand Synthesis System for RT-PCR kit; Invitrogen) with a mixture of pooled 20X TaqMan gene expression assay probes and 2X Taqman PreAmp Master Mix (Life Technologies, ThermoFisher, USA). Gene expression was analysed using a Fluidigm Dynamic array Biomark HD system (Fluidigm, USA). Genes were determined by relative expression calculated by change in cycle threshold (ΔCt) between each gene of interest and endogenous housekeeping gene *RPS32*. Levels of mRNA expression relative to geometrical average of house-keeping genes were determined using the 2^−ΔΔCT^ method [20] and expressed relative to the CTL group. Due to insufficient sampling, mRNA analysis of LPS lambs in the caudate could not be conducted.

### Statistical analysis

All statistical analyses were conducted using GraphPad Prism (version 10.2.1; GraphPad Software, CA, United States). Data were assessed for normality using the Shapiro-Wilk Test. Physiological parameters of animals at the start and end of experiment were compared using paired t-tests. For animal characteristics, RT-qPCR (grey and white matter) and immunohistochemical analyses, data sets were assessed for the main effects of LPS (*P*_*LPS*_), dopamine (*P*_*DA*_) and interactions between LPS and dopamine (*P*_*INT*_) by two-way ANOVA. Where a significant interaction was observed, post-hoc analysis was performed using Tukey’s multiple comparison test. For statistical analysis of RT-qPCR data in the caudate, a one-way ANOVA was conducted and significant F values were followed up with Tukey’s multiple comparison test. All mRNA expression from RT-qPCR analysis are expressed as relative change (2^−ΔΔCT^) and log10-transformed to maintain normal distribution for statistical analysis. Data are presented as mean ± SD and an alpha of 0.05 was considered statistical significance for all analyses.

**Table 1:** Genes of interest

Biological Process	Gene Name	ID	Taqman Code
Dopamine-related	Dopamine receptor D1	*DRD1*	Bt03223051_s1
	Dopamine receptor D2	*DRD2*	Oa04895884_m1
	Monoamine oxidase A	*MAOA*	Oa04709308_m1
	Monoamine oxidase B	*MAOB*	Oa03251801_mH
	Dopamine transporter	*SLC6A3*	Oa04675060_m1
Vasculature-related	Angiopoietin 1	*ANGPT1*	Oa04876148_m1
	Angiopoietin 2	*ANGPT2*	Oa04857533_m1
	Claudin-1	*CLDN1*	Oa03217991_m1
	Occludin	*OCLN*	Oa04728972_m1
	Vascular endothelial growth factor A	*VEGFA*	Oa04653812_m1
Oxidative stress	Myeloperoxidase	*MPO*	Oa04654413_g1
	NADPH oxidase 1	*NOX1*	Oa04709255_g1
	Superoxide dismutase 2, extracellular	*SOD3*	Oa04858164_m1
	Nitric oxide synthase 3	*NOS3*	Oa04907031_MH
Housekeeping genes	Ribosomal Protein L32	*RPL32*	Oa04893129_g1

## Results

### Lamb characteristics

Lamb characteristics (126–127 dGA) of the four groups are presented in Table 2. The lambs in all 4 groups were of similar weight. The male to female ratios were balanced but due to the small numbers for each group, sex differences could not be assessed between groups.

Arterial blood gases, pH, and other metabolites were assessed at the beginning of experiment following delivery and resuscitation of the lamb, and again at the end of experiment after ~ 3–4 h of gentle ventilation (Table 2). At the beginning of the experiment, there were no differences in pH, PaCO_2_, PaO_2_, HCO_3_^−^, and base excess between groups. pH, PaCO_2_, and PaO_2_, remained at similar levels by the end of experiment and remained similar across groups. At the end of experiment, lambs which received dopamine had increased HCO_3_^−^ and base excess, with the increase in HCO_3_^−^ greatest in LPS-exposed lambs (LPS vs. LPS_DA_: *P* = 0.008). Moreover, in CTL, CTL_DA_ and LPS lambs, HCO_3_^−^ concentrations had decreased by the end of experiment compared to the beginning of the experiment (*P* = 0.015, *P* = 0.008, *P* = 0.053 respectively). Haemoglobin concentrations were similar between the start and end of experiment within groups, but there was a difference between groups with LPS exposure lowering haemoglobin concentrations, whereas lambs allocated to the dopamine treatment had increased haemoglobin concentrations irrespective of the time of sampling (Table 2).

**Table 2:** Lamb characteristics and blood gas measurements at the beginning (after delivery and stabilisation with ventilation) and end of experiment

Groups	CTL	CTL_DA_	LPS	LPS_DA_	2-WAY ANOVA		
*n*	9	7	6	6	*P*_LPS_	*P*_DA_	*P*_INT_
**Gender (male/total)**	4/9	3/7	3/6	3/6			
**Weight (kg)**	3.16 ± 0.31	3.01 ± 0.46	3.44 ± 0.39	2.96 ± 0.45	0.445	0.057	0.296
**Parameter**	**Beginning of Experiment**						
**pH**	7.30 ± 0.11	7.31 ± 0.12	7.32 ± 0.04	7.28 ± 0.09	0.860	0.826	0.506
**PaCO**_**2 **_ **(mmHg)**	51.89 ± 13.51	55.26 ± 13.80	47.67 ± 8.09	61.10 ± 11.36	0.865	0.088	0.296
**PaO**_**2 **_ **(mmHg)**	96.58 ± 90.63	98.20 ± 38.60	88.67 ± 46.68	123.52 ± 30.64	0.722	0.459	0.499
**HCO**_**3**_^**− **^**(mmol.L**^**− 1**^)	25.16 ± 3.75	26.70 ± 3.00	24.20 ± 1.29	27.14 ± 1.16	0.822	0.062	0.545
**Base excess (mmol.L**^**− 1**^)	-1.11 ± 3.68	-0.29 ± 3.99	-2.13 ± 2.06	0.23 ± 2.89	0.849	0.232	0.560
**Haemoglobin (g.dL**^**− 1**^)	11.76 ± 1.79	13.33 ± 1.92	9.62 ± 1.41	12.17 ± 1.74	0.030*	0.008**	0.498
**Parameter**	**End of Experiment**						
**pH**	7.34 ± 0.12	7.37 ± 0.10	7.28 ± 0.08	7.31 ± 0.18	0.214	0.501	0.987
**PaCO**_**2 **_ **(mmHg)**	43.58 ± 12.82	42.60 ± 9.04	40.83 ± 8.47	57.28 ± 27.70	0.344	0.223	0.172
**PaO**_**2 **_ **(mmHg)**	59.74 ± 20.89	82.00 ± 31.27	97.67 ± 19.10	88.30 ± 33.40	0.052	0.554	0.155
**HCO**_**3**_^**− **^**(mmol.L**^**− 1**^)	21.19 ± 5.11^	23.69 ± 2.25^^	18.32 ± 4.34	25.90 ± 1.36##	0.821	0.002**	0.089
**Base excess (mmol.L**^**− 1**^)	-4.80 ± 6.29	-1.10 ± 3.57	-7.43 ± 5.20	-0.73 ± 4.22	0.566	0.014*	0.449
**Haemoglobin (g.dL**^**− 1**^)	11.73 ± 2.19	13.49 ± 1.71	8.90 ± 1.73	12.80 ± 1.60#	0.034*	0.002**	0.182

Data expressed as mean ± SD and analysed by 2-Way ANOVA; post hoc tests followed up with Tukey’s multiple comparisons test. Significant main effects are in bold and indicated as P*<0.05, P**<0.01; significant post-hoc comparison between LPS vs. LPS_DA_ are indicated as *P*#<0.05, *P*#<0.01. Blood gas measurements comparisons between beginning and end of experiment in each group were analysed using paired *t*-tests and paired significant differences are indicated as *P*^<0.05, *P*^^<0.01

Data expressed as mean ± SD and analysed by 2-Way ANOVA; post hoc tests followed up with Tukey’s multiple comparisons test. Significant main effects are in bold and indicated as P*<0.05, P**<0.01; significant post-hoc comparison between LPS vs. LPS_DA_ are indicated as *P*#<0.05, *P*#<0.01. Blood gas measurements comparisons between beginning and end of experiment in each group were analysed using paired *t*-tests and paired significant differences are indicated as *P*^<0.05, *P*^^<0.01.

### Vascular leakage and vascular remodeling are increased by LPS-exposure

**Fig. 1:**
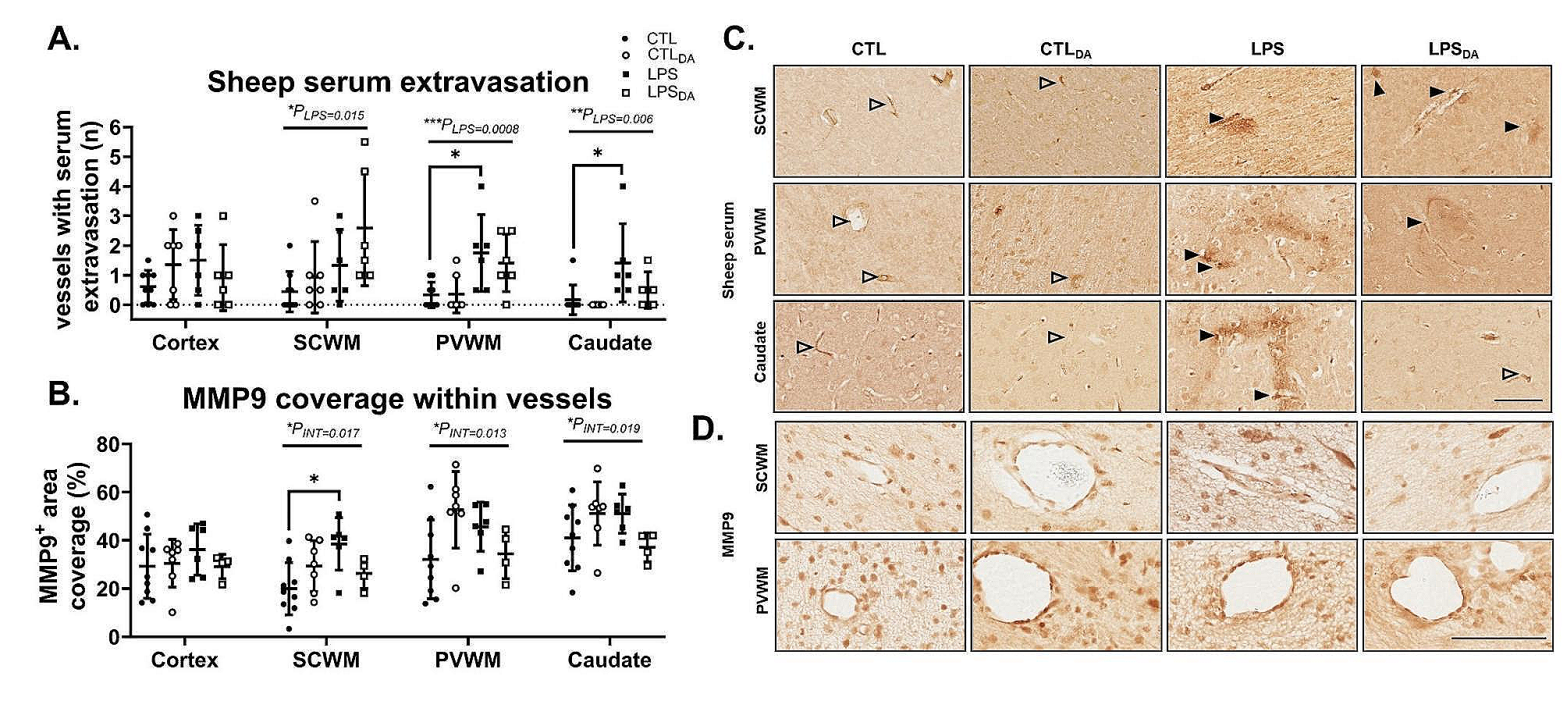
Sheep serum and MMP9 positive immunohistochemistry (**A**) Quantification of vessels with sheep serum immunopositive leakage and (**B**) quantification of MMP9 area coverage within vessels. Data are mean ± SD. Two-way ANOVA, *P < 0.05, **P < 0.01, ***P < 0.005. (**C**) Representative images of sheep serum extravasation from blood vessels into the brain parenchyma (black arrowhead), and sheep serum contained within the blood vessels (white arrowhead) in subcortical white matter (SCWM), periventricular white matter (PVWM) and caudate; and (**D**) representative images of MMP9 positive immunohistochemistry in the subcortical white matter (SCWM), periventricular white matter (PVWM). Scale bar represents 100 μm

Brain regions were assessed for vascular leakage as indicated by vessels surrounded by a halo of extravasated sheep serum (Fig. 1A&C). In the SCWM, PVWM and caudate regions, LPS-exposure increased the number of vessels with serum extravasation, with significant post-hoc differences between the groups not administered dopamine within the PVWM and Caudate (*P* = 0.019 and 0.017 respectively; Fig. 1A**&C**). There was no effect of LPS or dopamine on vascular leakage in the cortex.

Intra-amniotic LPS administration alone increased basement membrane breakdown and vascular remodeling within the SCWM, as indicated by the increase in area coverage of MMP9 immunolabelling in the control LPS lambs (*P* = 0.014; Fig. 1B**&D**). This effect was not observed in the dopamine-exposed lambs.

### Vasculature, astrocytic morphology and vessel coverage are altered by LPS-exposure and dopamine administration

**Fig. 2:**
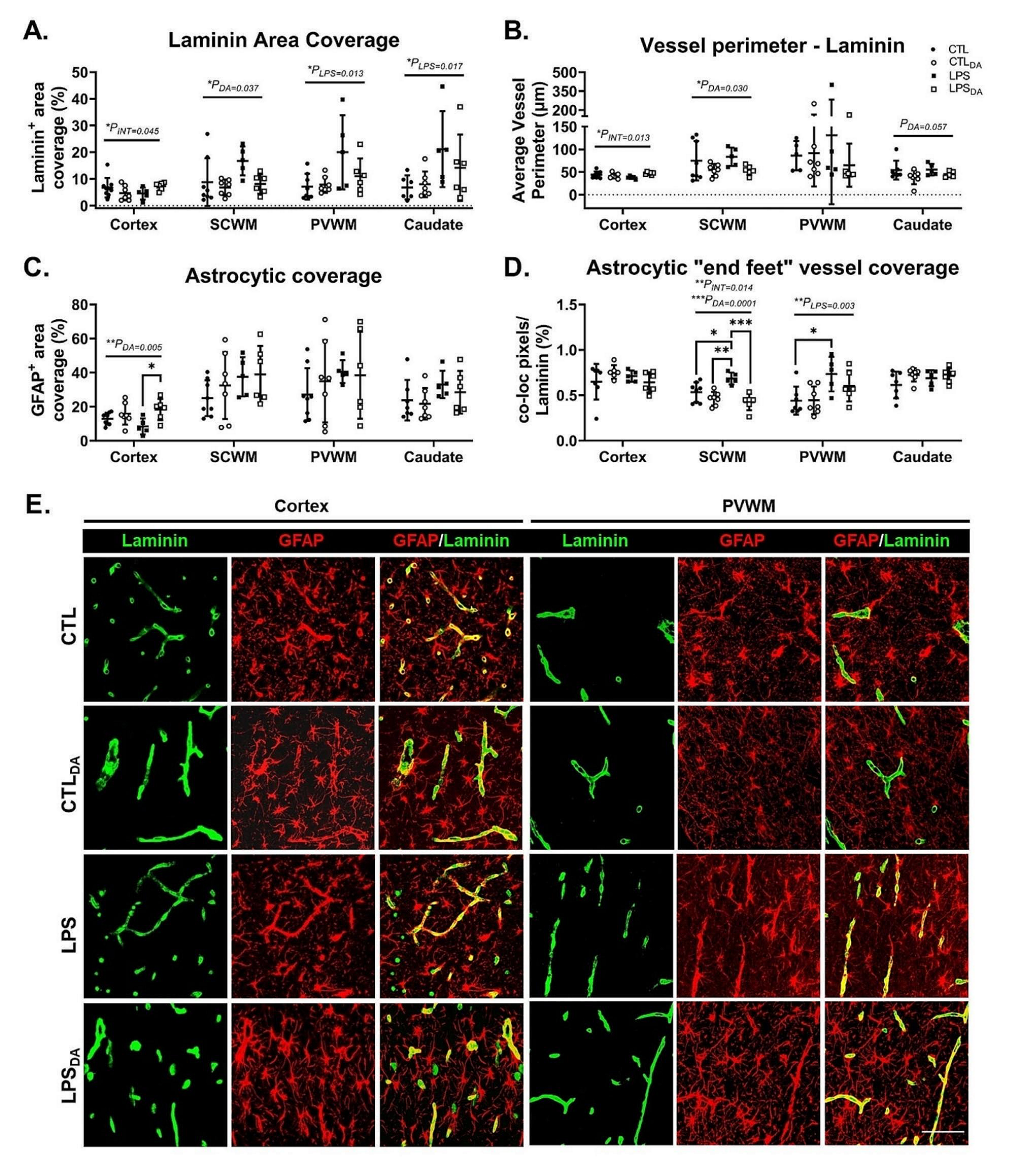
Laminin and GFAP positive immunohistochemistry (**A**) Quantification of laminin positive area coverage and (**B**) average vessel perimeter size as assessed using laminin positive immunolabelling. (**C**) Quantification of GFAP positive area coverage and (**D**) quantification of percentage of colocalization of GFAP with laminin positive immunolabelling. Data are mean ± SD. Two-way ANOVA, *P < 0.05, **P < 0.01, ***P < 0.005. (**E**) Representative images of laminin (green) and GFAP (red) positive immunofluorescent labellingand colocalization indicating astrocytic “end feet” covering of vessels in the cortical grey matter (cortex) and periventricular white matter (PVWM). Scale bar represents 50 μm

Assessment of the extracellular matrix protein laminin that lines endothelial cells was used as a surrogate measurement for vascular density and vessel size (Fig. 2A&B). LPS-exposure increased laminin expression, and therefore the calculation of vascular density, within the PVWM and caudate (Fig. 2A). In contrast, dopamine administration reduced the vascular density in the SCWM (Fig. 2A), and also decreased the overall vessel size within the SCWM (Fig. 2B).

Dopamine increased astrocyte coverage in the cortex, with the greatest effect occurring in the lambs that had been previously exposed to LPS (*P* = 0.021; Fig. 2C). No other effects on astrocyte coverage were observed.

Astrocytic “end feet” coverage on vessels was assessed by colocalization of GFAP- and laminin-immunoreactivity (Fig. 2D&E). LPS-exposure alone resulted in increased astrocytic vessel coverage in the SCWM and PVWM (*P* = 0.041 and *P* = 0.030 respectively). In contrast, dopamine in LPS-exposed lambs decreased astrocyte vessel coverage in the SCWM (*P* = 0.0005; LPS vs. LPS_DA_; Fig. 2D&E).

### Dopamine increases pericyte vascular coverage

**Fig. 3:**
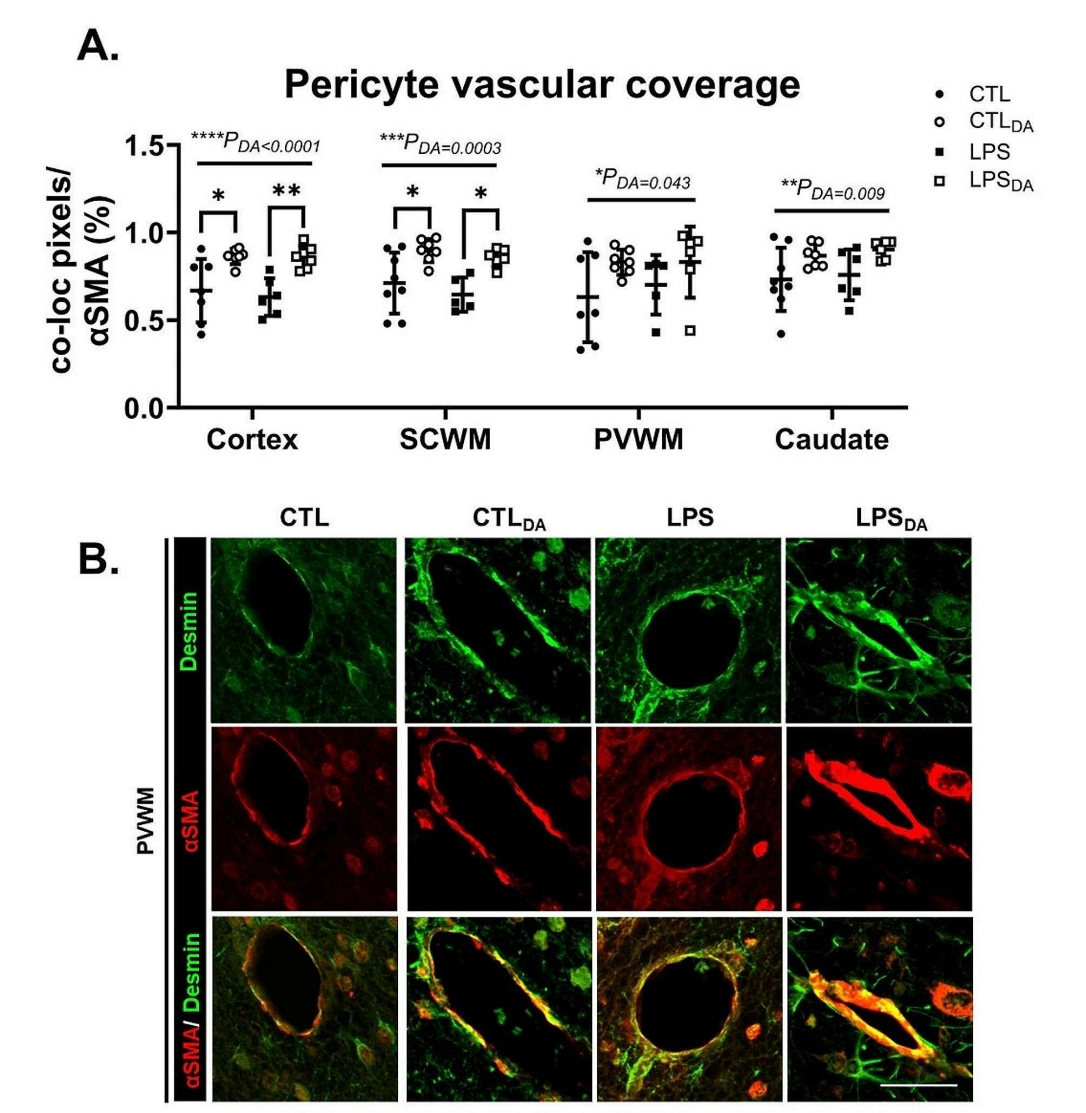
Desmin and αSMA positive immunohistochemistry (**A**) Quantification of percentage of colocalization of desmin with αSMA positive immunolabelling. Data are mean ± SD. Two-way ANOVA. (**E**) Representative images of desmin (green) and αSMA (red) positive immunofluorescent labellingand colocalization indicating pericyte coverage of vessels in the cortical grey periventricular white matter (PVWM). Scale bar represents 50 μm

Pericyte vascular coverage, considered a major factor in regulating capillary blood flow, was assessed using colocalization of vascular desmin and αSMA. Dopamine administration increased pericyte vascular coverage in all brain regions examined irrespective of LPS exposure (all *P*_DA_<0.05; Fig. 3A&B).

### Dopamine increases proliferative cells within vessel walls

**Fig. 4:**
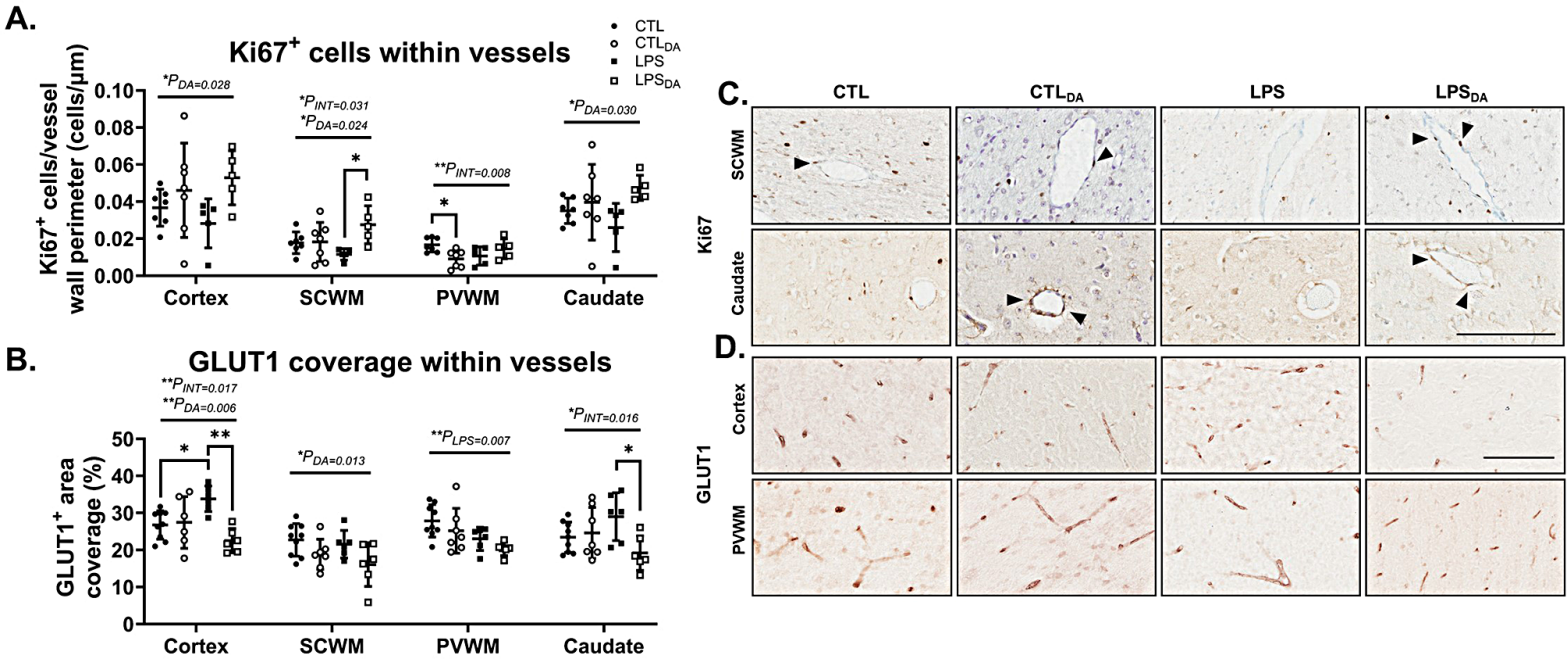
Ki67 and GLUT1 positive immunohistochemistry (**A**) Quantification of Ki67 immunopositive cells within vessel walls and (**B**) quantification of GLUT1 area coverage within vessel walls. Data are mean ± SD. Two-way ANOVA, *P < 0.05, **P < 0.01. (**C**) Representative images of Ki67 immunopositive cells (black arrowhead) within the blood vessels in the subcortical white matter (SCWM) and caudate, and (**D**) representative images of GLUT1 positive immunohistochemistry in the cortical grey matter (cortex) and periventricular white matter (PVWM). Scale bar represents 100 μm

Within the cortex, SCWM and caudate, dopamine administration increased vascular Ki67-immunopositive cells (Fig. 4A&C). In the SCWM specifically, the LPS lambs were most affected by dopamine-mediated increase in vascular Ki67-immunopositive cells (*P* = 0.026; Fig. 4A&C). In the PVWM of control lambs however, dopamine had an opposite effect and decreased Ki67-immunopositive cells within vessels (*P* = 0.031; Fig. 4).

### Dopamine decreases endothelial glucose transporter

Microvascular GLUT1 expression provides a marker for BBB maturity and function [21]. Dopamine decreased GLUT1 coverage in the SCWM in both control and LPS lambs (*P*_DA_=0.013), and also in the cortex and caudate in the LPS groups (*P* = 0.002 and 0.025 respectively) (Fig. 4B&D). In the PVWM, LPS-exposure decreased GLUT1 area coverage (Fig. 4B&D). Within the cortex, LPS-exposure increased GLUT1 area coverage, but this increase was not observed in the LPS-treated lambs after administration of dopamine (*P* = 0.043).

### Dopamine decreases coverage of neuronal dendrites

**Fig. 5:**
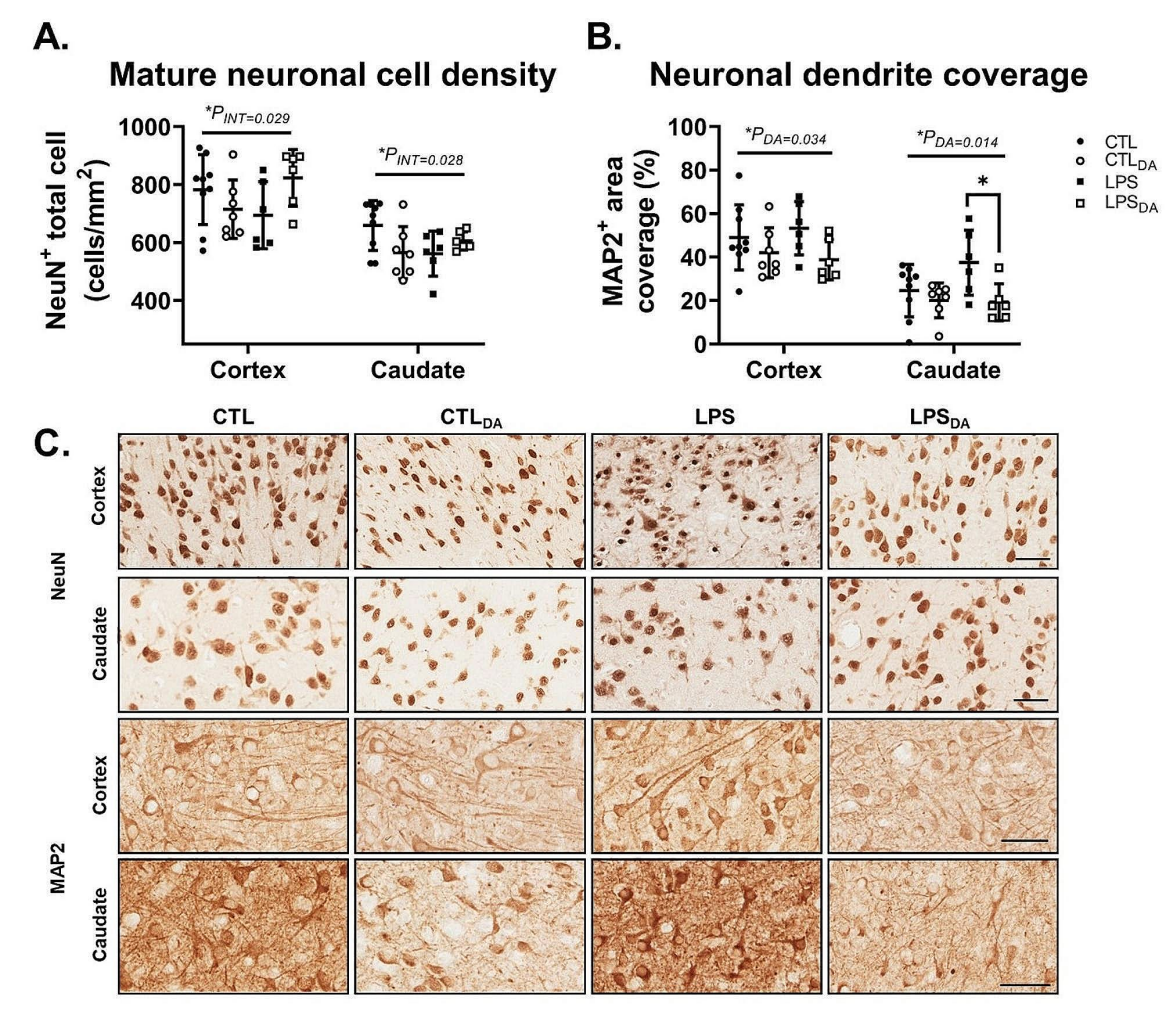
NeuN and MAP2 positive immunohistochemistry (**A**) NeuN immunopositive cell density indicating mature neuronal population and (**B**) MAP2 immunopositive area coverage indicating coverage of neuronal dendrites. Data are mean ± SD. Two-way ANOVA, *P < 0.05. (**C**) Representative images of NeuN- and MAP2-positive cells in the cortical grey matter (cortex; Layer V) and caudate. Scale bar represents 50 μm

Mature neuronal population and neuronal dendritic integrity within grey matter regions were assessed with NeuN and MAP2 immunolabelling respectively (Fig. 5). There was a significant interaction of LPS and DA on NeuN immunopositive neurons in the cortex and caudate, although post-hoc analysis did not identify significant differences between groups (Fig. 5A&C). Dopamine administration decreased neuronal dendrite coverage within both the cortex and caudate (both *P*_*DA*_<0.05; Fig. 5B&C). However, in the caudate specifically, the decrease in neuronal dendrite coverage with dopamine was greater in LPS-exposed lambs (*P* = 0.045; Fig. 5B&C).

### Effects of LPS and dopamine treatment on mRNA levels of genes relating to vasculature integrity, dopamine receptors/transporters, and markers of oxidative stress

#### Vascular integrity

**Fig. 6:**
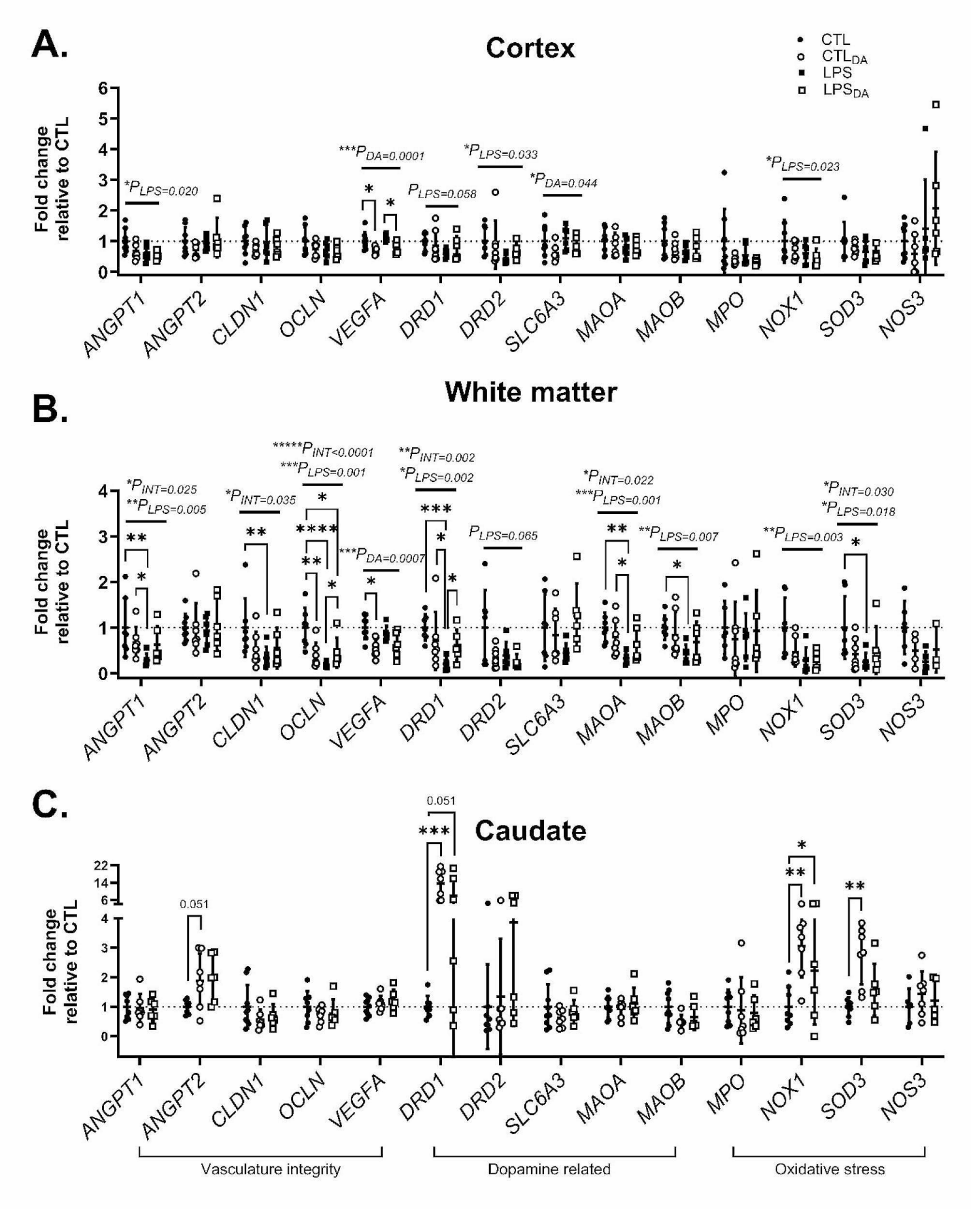
Fold change of mRNA levels mRNA expression of genes relating to vasculature integrity, dopamine, and oxidative stress measured within the (**A**) cortex, (**B**) combined white matter and (**C**) caudate. mRNA expression expressed relative to CTL. Data are mean ± SD. Cortex and white matter data analysed using two-way ANOVA, Tukey’s post-hoc analyses. Note mRNA levels in the caudate of LPS lambs were not conducted and data analysed using a one-way ANOVA, Tukey’s post-hoc analyses. *P < 0.05, **P < 0.01, ***P < 0.001, ****P < 0.0001

LPS exposure decreased angiopoietin *ANGPT1* (activator) mRNA levels in the cortex and white matter but did not affect expression of *ANGPT2* (agonist/antagonist) (Fig. 6A&B). LPS exposure also decreased expression of genes associated with formation of vascular tight junctions - *CLDN1* and *OCLN* – in the white matter of lambs not administered dopamine (*P* = 0.028 and < 0.0001 respectively; Fig. 6B). Dopamine decreased *VEGFA* mRNA expression in the cortex in both control and LPS groups (*P* = 0.027 and 0.009 respectively; Fig. 6A), and also in the control groups in the white matter (*P* = 0.012; Fig. 6B). Within the white matter, dopamine-administered lambs had decreased *OCLN* mRNA levels compared to CTL lambs (CTL vs. CTL_DA_, *P* = 0.004 and CTL vs. LPS_DA_, *P* = 0.030; Fig. 6B). In the caudate, dopamine showed a trend for upregulating *ANGPT2* mRNA levels in CTL_DA_ lambs compared to CTL lambs (*p* = 0.051; Fig. 6C).

#### Dopamine signalling

LPS exposure decreased dopamine receptors *DRD1* and *DRD2* mRNA levels in the cortex and white matter (Fig. 6A&B). In the white matter specifically, the LPS-mediated decrease in *DRD1* mRNA levels noted above was significantly less following dopamine administration, with mRNA levels higher in LPS_DA_ lambs compared to LPS only (*P* = 0.020; Fig. 6B). In the caudate, dopamine administration significantly increased the *DRD1* mRNA levels compared to CTL (*P* = 0.0002) with a trend for increase also occurring in LPS_DA_ lambs (*P* = 0.051; Fig. 6C). The mRNA levels of dopamine transporter SLC6A3 was decreased in dopamine administered lambs compared to non-dopamine administered lambs in the cortex (Fig. 6A). mRNA expressions of monoamine oxidases A and B, the key enzymes that metabolises dopamine, were unaffected by dopamine but were downregulated in LPS lambs compared to CTL lambs in the white matter (*P* = 0.001 and 0.018 respectively; Fig. 6B).

#### Oxidative stress

LPS decreased the mRNA levels of *NOX1* (Fig. 6A) in the cortex and white matter. In the white matter, LPS decreased *SOD3* mRNA levels in lambs not administered dopamine (*P* = 0.011; Fig. 6B). Dopamine increased both *NOX1* and *SOD3* mRNA levels in the caudate, with *NOX1* expression also increased in the LPS_DA_ compared to CTL lambs (Fig. 6C).

## Discussion

Intrauterine inflammation is a common precursor to preterm brain injury, and has often been associated with impaired brain development and long-term neurodevelopmental abnormalities [22]. Infants exposed to infection and inflammation often suffer from cardiovascular dysfunction including hypotension, and are therefore treated with dopamine infusion to restore blood pressure. To our knowledge, this is the first study on the combined and separate impacts of intrauterine inflammation and dopamine infusion on the preterm NVU, including histological assessments of the NVU, and the transcriptional changes of genes associated with the impact of inflammation and dopamine. Overall, the effects of LPS and dopamine on the NVU were either independent or opposite in direction, and we found no compounding or synergistic effects of LPS and dopamine. Exposure to intrauterine inflammation was associated with increased vascular leakage in the presence of increased vessel density, increased astrocytic “end-feet” vascular coverage, basement membrane breakdown and vascular remodeling mainly within the white matter, with downregulated gene expression for tight junctions also in the white matter. On the other hand, dopamine administration decreased vessel density and size, downregulated VEGF gene expression, and increased pericyte vascular coverage, but it also decreased astrocytic “end-feet” vascular coverage despite an overall higher astrocytic coverage. In addition, dopamine decreased glucose transporter and barrier function with downregulation of the *OCLN* tight junction gene, and decreased neuronal dendrite coverage. As expected, dopamine upregulated genes for dopamine receptors, and for markers of oxidative stress in the caudate. Taken together, this study demonstrates that components of the preterm NVU are disrupted by intrauterine inflammation and are also sensitive to the presence of increased levels of dopamine.

### The effects of LPS exposure on the preterm NVU

#### LPS and vessels/BBB

Disturbances of the NVU physiology and morphology have been identified as one of the leading causes of neurodevelopmental disorders [23]. We have previously demonstrated in preterm lambs that exposure to intrauterine inflammation results in a negative cerebral functional haemodynamic response following somatosensory stimulation [8]. This negative haemodynamic response suggested impaired neurovascular coupling, possibly reflecting cellular changes at the level of the NVU. Our findings of an increase in cerebral vascular leakage after LPS exposure associated with an increased vessel density and astrocytic “end-feet” vascular coverage suggests disrupted structure of the NVU, possibly leading to functional deficits. Studies in adult rats have shown that disrupted endothelial cells in the pial arteries disturb the stimulus-evoked vasodilation associated with neurovascular coupling [24]. The increases in MMP9 expression associated with the LPS treatment is suggestive of vascular remodeling, and the decreased expression of the tight junction proteins (*CLDN1, OCLN*) further support vascular and BBB disruption in the white matter [25, 26]. Such inflammation-induced BBB permeability and leakage has previously been associated with preterm white matter brain injury in the sheep fetus [27–29].

#### LPS and astrocytes

Astrocytes are a key component of the NVU, and they modulate the vasculature interface and the BBB [30]. In our study, LPS exposure increased astrocytic end-feet vessel coverage in the white matter, but not the overall astrocytic coverage. This result is in contrast to a previous study conducted in fetal sheep at a younger gestation (~ 104–108 dGA) where LPS was administered as a chronic LPS infusion and boluses over 5 days, resulting in decreased astrocytic end-feet vessel coverage, increased astrocytic coverage, and reduced microvascular vessel density [31]. The differences in astrocyte and vasculature morphology may reflect the study’s younger gestation, the chronic LPS regimen and/or the timing of brain assessment. For example, the disruption to the NVU may arise from both direct cytokine actions and secondary cytokine production in the fetal brain by activated glial cell such as astrocytes [32]. This cytokine induction differs in cases of repeated and acute LPS challenges [33]. We cannot ascertain whether the increased astrocytic end-feet coverage contributes to increased BBB leakage; or the BBB breakdown and inflammation induces increased astrocytic end-feet coverage. We speculate that, firstly, the increased astrocytic end-feet coverage post-LPS exposure is dysfunctional or ineffective, thus causing increased BBB leakage [34]. Alternatively, the increased astrocytic end-feet coverage is a response to the BBB leakage following LPS exposure [35]. Regardless, the increased astrocytic end-feet vessel coverage highlights injury to the NVU and likely its dysfunction.

#### LPS and dopamine genes

We found that LPS exposure downregulated gene expression for dopamine receptors D_1_ and D_2_, and monoamine oxidase A and B. This is the first study to identify a molecular modulation of dopamine signaling pathways in the preterm brain due to intrauterine inflammation. Other studies have indicated the association of prenatal inflammation, developmental perturbation of the dopaminergic system, and the eventual development of neuropsychiatric disorders such as schizophrenia [36, 37]. Thus, the transcriptional dysregulation of the dopamine pathway provides a possible mechanistic link between prenatal inflammatory events and neurodevelopmental disorders.

### The effects of dopamine administration on the preterm NVU

#### DA and vessels/BBB

Dopamine infusion itself resulted in morphological changes mainly to the cerebral microvasculature. Firstly, dopamine decreased vessel density and size in the white matter suggesting a cerebrovascular vasoconstrictive response, as previously observed in hemodynamically stable unanesthetized preterm fetal sheep treated with dopamine [38]. It is important to note that our lambs were not hypotensive prior to dopamine treatment. Therefore, the dopamine-induced vasoconstriction could reflect an autoregulatory response to the increase in systemic blood pressure [14], and/or the dominant activation of α-adrenergic receptors in the immature cerebral vasculature [38].

Secondly, pericyte vascular coverage was increased following dopamine exposure in both control and LPS lambs. Pericytes promote vascular structural stability and protect the brain from blood-borne elements and bleeding. Notably, dopamine has a putative role in pericyte-vascular-neuronal function [39, 40]. Endogenous dopamine mediates pericyte relaxation [40, 41]; and exogenous dopamine promotes mature pericyte vascular coverage via the D_2_ receptors [42]. However, we also found that exogenous dopamine attenuated GLUT1 expression and downregulated *OCLN* tight junction gene expression, suggesting disruption of the BBB and its function. Of note, the reduced GLUT1 coverage within vessel walls may be secondary to the decreased vessel size and reflect vasoconstriction following the dopamine infusion. In a similar respect, the decreased vessel size may also explain the increase in Ki67^+^ cell density per vessel perimeter of dopamine infused lambs, because the smaller vessel perimeter results in a higher Ki67^+^ cell density. Studies have also shown that exogenous dopamine crosses the preterm BBB, although it remains unclear if the increased BBB permeability was due to prematurity or induced by dopamine [43, 44]. The actions of exogenous dopamine on the preterm BBB and vasculature require further investigations.

#### DA and astrocytes

Dopamine infusion was associated with astrogliosis in the cortex as indicated by the increased overall coverage of GFAP immuno-positive labelling, whereas there was attenuated astrocytic vessel coverage in the white matter. The increased cortical GFAP coverage may be due to changes in astrocyte morphology as dopamine exposure to cultured astrocytes can increase the growth of astrocytic processes growth (> 200% increase), although this does not necessarily mean an increased interaction with the vasculature [45]. Indeed, while LPS increased astrocytic vessel coverage in the white matter, addition of dopamine reversed this change (Fig. 2D) which may indicate a retraction of the astrocytic processes on the vasculature. Interestingly, there was no exacerbation of vascular leakage in the LPS_DA_ lambs which may be a result of the overall cerebrovascular constriction, or the increased pericyte coverage caused by dopamine treatment. In addition, the *VEGFA* mRNA expression in both grey and white matter was downregulated after dopamine exposure, which could affect the vascular–astrocyte interactions as VEGF is closely associated with astrocyte proliferation [46], and astrocyte-derived VEGF promotes vascular growth and development [47].

#### DA and neuronal dendrites

Dopamine decreased neuronal dendritic coverage within grey matter regions, consistent with in vitro observations of primary cortical neuronal cultures where dopamine receptor activation decreased dendritic extension via increased MAP2 phosphorylation [48]. Disturbance to neuronal dendritic development and increased astrogliosis with dopamine infusion may have compounded effects on the already vulnerable neuronal network maturation that is known to be present in preterm infants [49].

#### DA and dopamine genes

In the caudate, dopamine administration upregulated the *DRD1* gene which encodes for the dopamine receptor D1, and also upregulated *NOX1* and *SOD3* genes which are involved in mediating oxidative stress. Major dopaminergic pathways with high densities of dopaminergic receptors are found in the basal ganglia [50]. Our findings suggest that increased extracellular dopamine and its degradation may lead to elevated levels of oxidative metabolites [51], given that NOX catalyses the production of reactive oxygen species and that SOD3 in an antioxidant enzyme. Future studies are needed to fully elucidate the long-term effects on the dopaminergic systems at cellular and functional levels in the preterm brain after exposure to exogenous dopamine.

### Clinical implications

This study has revealed an important interaction between intrauterine inflammation and postnatal exposure to dopamine, with significant and regional effects on neurovascular structure in the preterm lamb. The NVU maintains brain homeostasis by facilitating neurovascular coupling and the trafficking of blood-borne molecules and cells into and out of the brain. Alterations to the NVU due to intrauterine inflammation has long been thought to be the basis of long-term neurological disabilities [52, 53], including neuropsychological disorders such as autism [54] and schizophrenia [55] in which NVU impairments have been identified [56, 57]. BBB breakdown may also be an early biomarker for later cognitive dysfunction [58]. Importantly, we showed that a treatment commonly used to support the hypotensive preterm infant - intravenous dopamine - is likely to have additional effects on the NVU already compromised by exposure to inflammation. Future research should aim to understand the impact and interactions of other medical interventions on the preterm NVU after intrauterine inflammation, such as, for example, antenatal and postnatal steroid treatments, given that the glucocorticoid receptor is important for the stabilization of vascular endothelial cells and proliferation of neural stem/progenitor cells [59]. Protection of the NVU could therefore be a potential therapeutic target to reduce long-term neurodevelopmental deficits in preterm infants.

### Strengths and limitations

Sheep have been used extensively to study the preterm cerebral circulation and neuropathies [60]. The preterm lamb at 126–127 dGA (0.8 gestation) displays oligodendrocyte maturation similar to a late preterm human baby of ~ 35 weeks, and also cerebral vascular pathophysiology and immature neurovascular coupling comparable to a preterm human baby [61, 62], and is thus an excellent model for the study of clinical conditions and therapies to assess their effects in the preterm brain. Our model simulated clinical management for preterm delivery following intrauterine inflammation. However, the preterm lambs in this study were normotensive rather than hypotensive, and received dopamine infusion for a relatively brief duration of 90 min [8, 14], which may have limited our findings of the impact of dopamine. Also, we were able to characterized the NVU at only a single timepoint at 3–4 h after birth. Assessment at later time-points in a maturing brain would delineate the further developmental impact on the NVU. We assessed transcriptional changes within whole brain regions which contain a mixture of cellular populations; further insights using single cell transcriptomics would confirm the exact cell populations with the major transcriptional changes found in this study. Importantly, we demonstrate that both intrauterine inflammation and postnatal dopamine exposure independently disrupt the NVU which is a critical component in the pathophysiology of preterm brain injury.

## Conclusion

We report the cellular and molecular impact of intrauterine inflammation and dopamine, together and separately, on the preterm NVU. LPS exposure predominately resulted in vascular leakage in the presence of increased vessel density and vascular remodelling; while dopamine infusion resulted in decreased vascular density and size with increased pericyte coverage, but decreased astrocytic vascular coverage and integrity of the blood-brain barrier. Notably, we did not find any synergistic effects of LPS and dopamine, and we speculate that they may contribute independently to neurovascular disruption which could predispose the brain to further developmental impairments.

### Electronic supplementary material

Below is the link to the electronic supplementary material.

**Supplementary Fig. 1.** Schematic indicating fields sampled for histological assessment
Field of view (FOV) indicated in red boxes were sampled for assessment of cortical grey matter (cortex; yellow), subcortical (SCWM; green) white matter regions within the first, second, third and fourth parasagittal gyri and FOVs also assessed in the periventricular white matter (PVWM; pink) and caudate (blue).

## Data Availability

Data is provided within the manuscript or supplementary information files. The datasets used during the current study are available from the corresponding author upon reasonable request.

## Abbreviations

%: percent
α-SMA: alpha-smooth muscle actin
µm: micrometer
µg: microgram
ANOVA: analysis of variance
BBB: blood-brain barrier
CAT#: catalogue number
CBF: cerebral blood flow
CLDN1: claudin 1
CTL: control
DA: dopamine
DAT: dopamine transporter
dGA: days of gestation
dH_2_O: deionized water
dL: decilitre
DRD1: Dopamine receptor D1
FOV: fields of view
g: grams
GFAP: Glial fibrillary acidic protein
GLUT1: glucose transporter 1
GM: grey matter
h: hour
HCO_3_^−^: Bicarbonate
HR: heart rate
IBA-1: ionized calcium binding adaptor molecule-1
IgG: Immunoglobulin G
kg: kilogram
L: litre
LPS: lipopolysaccharide
M: moles
MABP: mean arterial blood pressure
MAP2: microtubule-associated protein 2
mg: milligram
min: minute
ml: millilitre
mm: millimetre
mM: millimole
mmHg: millimetres of mercury
MMP9: matrix metallopeptidase 9
mRNA: messenger ribonucleic acid
*n*: number of
NeuN: neuronal nuclei
NVU: neurovascular unit
OCLN: occludin
PaCO_2_: partial pressure of arterial carbon dioxide
PaO_2_: partial pressure of arterial oxygen
PVWM: periventricular white matter
RNA: Ribonucleic acid
RT-qPCR: Reverse transcription-quantitative polymerase chain reaction
SCWM: subcortical white matter
SD: standard deviation
SpO_2_: peripheral oxygen saturation
VEGFA: Vascular Endothelial Growth Factor A
WM: white matter
