# Supplementary material for: Intrauterine inflammation and postnatal intravenous dopamine alter the neurovascular unit in preterm newborn lambs
Source: J Neuroinflammation. 2024 May 28;21:142. doi: 10.1186/s12974-024-03137-0 (PMC11134744; doi:10.1186/s12974-024-03137-0)
Supplement: Supplementary file 1 — Supplementary Fig. 1. Schematic indicating fields sampled for histological assessment Field of view (FOV) indicated in red boxes were sampled for assessment of cortical grey matter (cortex; yellow), subcortical (SCWM; green) white matter regions within the first, second, third and fourth parasagittal gyri and FOVs also assessed in the periventricular white matter (PVWM; pink) and caudate (blue). [file 12974_2024_3137_MOESM1_ESM.docx]

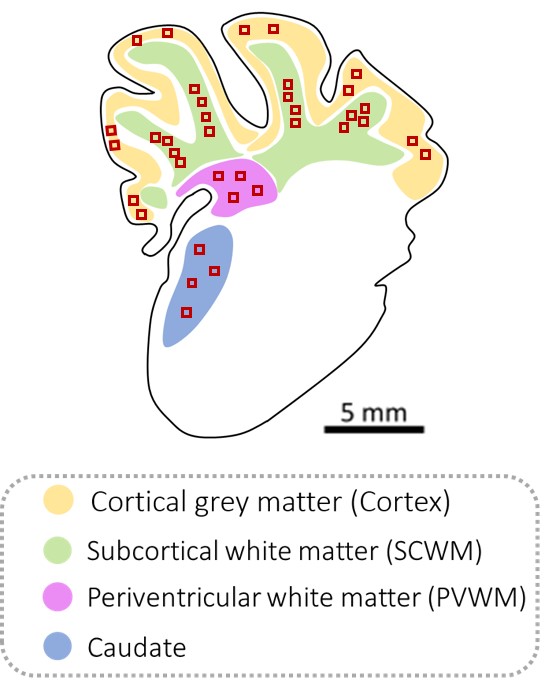


**Supplementary Figure 1. Schematic indicating fields sampled for histological assessment.**

Field of view (FOV) indicated in red boxes were sampled for assessment of cortical grey matter (cortex; yellow), subcortical (SCWM; green) white matter regions within the first, second, third and fourth parasagittal gyri and FOVs also assessed in the periventricular white matter (PVWM; pink) and caudate (blue).
